# 支气管内超声技术在胸部肿瘤中的应用现状和前景

**DOI:** 10.3779/j.issn.1009-3419.2010.05.02

**Published:** 2010-05-20

**Authors:** 拴盈 杨, 丽娜 卜

**Affiliations:** 1 710004 西安，西安交通大学医学院第二附属医院呼吸科 Department of Respiratory Medicine, the Second Hosipital Affiated to Xi'an Jiaotong University College of Medicine, Xi'an 710004, China; 2 710004 西安，西安市第二人民医院内科 Department of Medicine, the Second People's Hosipital of Xi'an, Xi'an 710004, China

支气管内超声（endobronchial ultrasound, EBUS）技术于1992年首次应用于临床^[1]^。随着超声及相关技术的进步，EBUS仪器性能日臻完善，操作技术日渐成熟，逐步建立了气道和纵隔的超声声像图谱，使支气管镜的检查范围从管腔内扩展到了管腔外，明显提高了支气管镜检查的效率，因而在呼吸系统疾病中的应用越来越广泛，迄今已成为胸部肿瘤诊断和治疗中最有前景的微创技术之一。

微型超声探头是EBUS最主要的组成部分。探头外带水囊使探头能更好地接触气道粘膜，获得更清晰的超声图像。根据超声探头的不同，将EBUS分为两种类型。一种为早期的EBUS，带直径2 mm的径向探头（radial probe, RP），分辨率为0.1 mm，可通过活检通道进入气道内进行360°成像扫描，可清楚地显示气道壁及其周围组织的细微结构，可显示深达5 cm的肺门及纵隔淋巴结，主要用于检查肺外周（亚段以下支气管）病变、判断肿瘤浸润管壁的深度、鉴别气道壁内病变的良恶性，但该探头不能进行实时监控下病灶活检。另一种EBUS为近年开发的凸面探头（convex probe, CP），该探头和镜体远端融合，顶端外径6.9 mm，扫描范围50°，可在超声实时引导下进行病灶活检，超声图像可以定格，可测量病灶的平面大小，获得支气管镜图像，该系统还有多普勒模式，可观察病灶的血供及其周围血管情况，该技术是近年来肺癌诊断和分期方法中最重要的进展之一，主要用于大气道壁及其周围组织病灶的观察和活检^[2, 3]^。

## 1 EBUS引导下经支气管针吸活检（EBUS transbronchial needle aspiratory, EBUS-TBNA）

一般情况下，只要超声支气管镜能够到达并能清楚显示的病灶，均可进行EBUS-TBNA。

纵隔镜是纵隔淋巴结检查的“金标准”，但其需在全身麻醉下进行，创伤大、风险高、费用贵，重复进行纵隔淋巴结活检几乎不可能。常规TBNA操作简单、创伤小、费用低，但多属于“盲穿”，不同研究^[4]^显示诊断准确率差异很大（20%-89%），其原因可能与淋巴结的大小、位置、操作者的熟练程度有关，而EBUS-TBNA不仅具有操作简单、微创等优势，而且定位更加精确，可以发现直径为2 mm-3 mm的淋巴结，显著提高了穿刺的准确性和安全性，其穿刺成功率不受淋巴结大小、位置的影响，可用于治疗后复查。Herth等^[5, 6]^报道EBUS-TBNA判断肺癌纵隔淋巴结转移的敏感性、特异性及阴性预测值分别为94%、100%和96%。Yasufuku等^[7]^比较了EBUS、计算机体层扫描（computed tomography, CT）及正电子发射计算机断层显像仪（positron emission tomography, PET）在96例确诊和6例影像学疑诊的肺癌患者的淋巴结分期中的价值。结果显示，CT、PET及EBUS诊断纵隔和肺门淋巴结转移的敏感性分别为77%、80%和92%，特异性分别为55%、70%和100%，准确率分别为61%、73%和98%。提示EBUS在确定肺癌淋巴结分期方面比CT及PET更具优势，尤其在淋巴结直径＜10 mm时。由于解剖结构的限制，EBUS-TBNA无法对第5、6、8、9组淋巴结进行活检^[8]^。Herth等^[9]^认为EBUS联合内镜超声（endoscopic ultrasonography, EUS）实时引导可以对几乎所有纵隔淋巴结进行活检。提示EBUS联合EUS显著降低了纵隔镜检查在纵隔淋巴结活检中的必要性，减少了肺癌淋巴结分期检查中的侵入性操作，有可能成为肺癌术前分期的新的“金标准”。

肺部周围型病灶，尤其是孤立肺结节（solitary pulmonary nodule, SPN）的定性诊断一直严重困扰着临床医师。通过可弯曲支气管镜进行经支气管肺活检（transbronchial biopsy, TBB）及支气管刷检（bronchial brushing, BB）被认为是诊断肺部周围型病灶的“金标准” ^[10]^。但TBB需在X线透视引导下进行；检查结果受病变大小、病变距肺门的距离的影响；对于＜2.5 cm的病变，诊断率仅为30%。Paone等^[11]^将221例肺部周围型病灶随机分为两组，分别进行EBUS-TBB（97例）和常规支气管镜下TBB（124例），结果发现，对于肺癌患者，当病灶＞3 cm时，两种方法的敏感性（分别为83%和77%）和准确性（分别为88%和84%）无明显差异，而当病灶＜3 cm时，EBUS-TBB的敏感性和准确性分别为75%和83%，显著高于TBB（分别为31%和50%）。提示EBUS可能是周围型肺癌早期诊断的强有力工具，尤其当病灶＜3 cm时。Chao等^[10]^对182例肺部周围型病灶进行了前瞻性随机研究，其中94例患者接受了EBUS-TBB及BB联合检查，88例接受了EBUS-TBNA、TBB及BB联合检查。结果显示，三种方法联合应用时诊断准确性为78%，两种方法联合应用时诊断准确性为61%，差异有统计学意义（*P*=0.015）；三种方法中TBNA准确性最高（63%），TBB为49%，BB仅为20%；探头位于病灶内时TBNA阳性率为64%，临近病灶时为59%，无统计学差异，探头位于病灶内时TBB和BB的阳性率分别为60%和24%，临近病灶时分别为23%和9%，差异均有统计学意义（P值分别为＜0.001和0.05）。提示三种方法联合检查效果最好；TBNA阳性率不受探头位置的影响。Kurimoto等^[12]^应用导管鞘在RP-EBUS引导下对肺周围型病灶进行了活检。对照病理学结果，作者根据超声图像内部结构（包括内部回声、血管和支气管是否通畅、高回声区域的形态）将病灶分为3个类型：Ⅰ型为均质型，Ⅱ型为点状或弧线型强回声，Ⅲ型为异质型。其中92%的Ⅰ型为良性疾病，99%的Ⅱ型和Ⅲ型为恶性疾病。李静等^[13]^认为超声图像中病灶边界清晰、内部低回声、回声不均匀、无点线状高回声及邻近血管移位、狭窄或中断征象对诊断周围型肺癌有一定价值。

CP-EBUS可以对邻近气道的肿瘤甚至肺尖肿瘤进行实时活检。由于纵隔结构复杂，进行有创检查风险较大，纵隔肿瘤的细胞学诊断比肺癌患者淋巴结转移的诊断要困难得多，但应用RP-EBUS可以准确定位纵隔病变并进行活检。但事实上，迄今在EBUS引导下进行纵隔肿瘤活检的报道尚少。

## 2 EBUS可清晰显示气道壁、纵隔及其周围组织的细微结构

EBUS显示支气管壁为7层结构：从管腔内面到外膜分别为粘膜（高回声）、粘膜下层、软骨层内面（高回声）、软骨层（低回声）、软骨层外面（高回声）、结缔组织（低回声）、外膜（高回声）。通过对粘膜下超声解剖结构的观察，EBUS可发现CT和支气管镜不能发现的气管内肿瘤；应用不同波长的超声，EBUS可以清楚地显示粘膜几乎完整的肿瘤粘膜下浸润（原位癌）^[14]^；腔内生长或管壁内的肿瘤一般以粘膜下层是否增宽、回声是否均匀、软骨和气管支气管外膜是否完整来判断肿瘤是否侵犯管壁及侵犯深度。Herth等^[15]^发现，以气道壁层状结构破坏作为诊断恶性肿瘤的标准，与病理诊断相比，EBUS诊断恶性肿瘤的准确率性达97%，明显高于自荧光支气管镜（69%）；EBUS可判断肿瘤是否累及纵隔、主动脉、腔静脉、肺动脉及其大分支，其诊断肺门淋巴结侵犯肺动脉的准确率可达94%；可分辨纵隔肿瘤和气管、支气管原发性肿瘤，尤其是判断实体瘤外压管壁还是侵润管壁；可诊断食管癌有无气管支气管侵犯，准确率＞90%，显著优于CT（准确率为60%左右）。

## 3 EBUS的安全性

EBUS是一项很安全的临床检查。一般可门诊进行。主要不良事件有：自限性出血，个别情况下有中量出血（30 mL-60 mL）^[16]^；对左下叶支气管的操作可出现房性心动过速等心律失常，但均为自限性，无严重后果；2%的患者出现气胸^[17]^。操作技术熟练时，EBUS检查肿瘤侵犯深度一般需3.5 min，EBUS-TBNA需6.3 min，EBUS-TBB需（8.8±0.8）min^[16]^。

## 4 存在问题及注意事项

由于操作过程中超声图像不断变化，故分析超声图像较难，要求操作医师必须对气管、支气管及纵隔结构非常熟悉，才能熟练分析超声图像；EBUS管径较粗，需经口插入，故不能取代常规支气管镜；TBNA时穿刺针往往难以与气道壁垂直，选择穿刺点时应考虑到这一因素；由于EBUS-TBNA取材少，多为细胞病理学诊断，故对纵隔淋巴结上皮性癌转移的判断价值更高，对于间叶来源的肿瘤如淋巴瘤及良性疾病如结核、真菌感染等的诊断需临床和病理科医师密切配合；在EBUSTBNA操作中，穿刺针应在淋巴结内有足够的抽送次数（10次以上），以提高阳性率^[3, 18]^；EBUS可分辨淋巴滤泡和淋巴窦细微结构，但无可靠征象判断肿大的淋巴结是否存在转移；应用EBUS判断肺部周围型肿瘤是否侵犯软骨可能出现假阳性。

## 5 应用前景

由于EBUS仪器精密，价格昂贵又易于损坏，维修费用也同样昂贵，故该技术目前远未普及；迄今关于EBUS-TBNA对周围型肺部病灶的诊断价值的研究尚少，且多为回顾性研究，或小样本研究，严格的前瞻性随机对照研究目前尚未见报道^[10]^；对EBUS在诊断癌前病变中的价值的研究鲜见。尽管如此，已有的研究提示：EBUS检查操作简单，安全性好，在肺门和纵隔淋巴结、早期气管内肿瘤（原位癌）、SPN及肺癌分期中较其它方法有明显的优势，拓展了气管镜的应用范围。相信随着超声及相关技术的进一步发展，EBUS必将在胸部疾病诊治中发挥越来越大的作用。
